# A Prefiltered Cuckoo Search Algorithm with Geometric Operators for Solving Sudoku Problems

**DOI:** 10.1155/2014/465359

**Published:** 2014-02-23

**Authors:** Ricardo Soto, Broderick Crawford, Cristian Galleguillos, Eric Monfroy, Fernando Paredes

**Affiliations:** ^1^Pontificia Universidad Católica de Valparaíso, 2362807 Valparaíso, Chile; ^2^Universidad Autónoma de Chile, 7500138 Santiago, Chile; ^3^Universidad Finis Terrae, 7501015 Santiago, Chile; ^4^CNRS, LINA, University of Nantes, 44322 Nantes, France; ^5^Escuela de Ingeniería Industrial, Universidad Diego Portales, 8370109 Santiago, Chile

## Abstract

The Sudoku is a famous logic-placement game, originally popularized in Japan and today widely employed as pastime and as testbed for search algorithms. The classic Sudoku consists in filling a 9 × 9 grid, divided into nine 3 × 3 regions, so that each column, row, and region contains different digits from 1 to 9. This game is known to be NP-complete, with existing various complete and incomplete search algorithms able to solve different instances of it. In this paper, we present a new cuckoo search algorithm for solving Sudoku puzzles combining prefiltering phases and geometric operations. The geometric operators allow one to correctly move toward promising regions of the combinatorial space, while the prefiltering phases are able to previously delete from domains the values that do not conduct to any feasible solution. This integration leads to a more efficient domain filtering and as a consequence to a faster solving process. We illustrate encouraging experimental results where our approach noticeably competes with the best approximate methods reported in the literature.

## 1. Introduction

**Figure 1:**
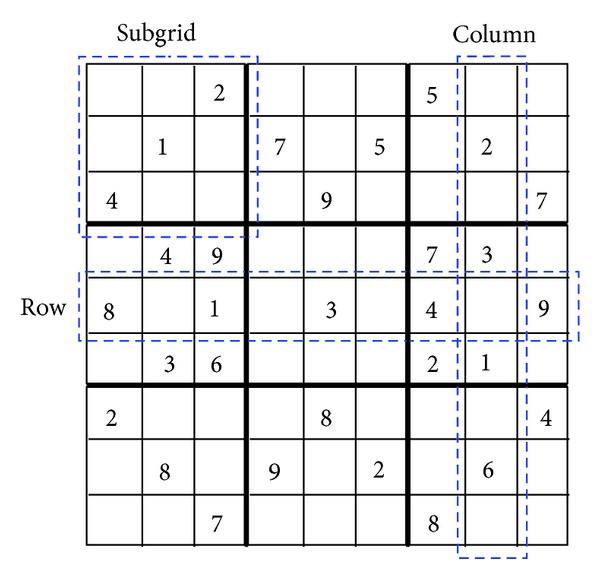
Sudoku puzzle instance.

The Sudoku is a logic-based placement puzzle, widely present as pastime game in newspapers and magazines. It was initially popularized in Japan during the eighties, but today it is a worldwide popular game and a useful benchmark for testing artificial intelligence solving techniques. A Sudoku puzzle consists in filling a board of 9 × 9 subdivided into subgrids of size 3 × 3 so that each row, column, and subgrid contains different digits from 1 to 9. A Sudoku problem includes prefilled cells, namely, the “givens,” which cannot be changed or moved (see Figure 1). Certainly, the amount of givens has limited or no impact on the difficulty of the problem. The difficulty is mostly dependent on the positioning of the givens along the puzzle. A useful difficulty classification including easy, medium, and hard Sudokus has been proposed by Mantere and Koljonen [1].

The literature reports various approaches to solve Sudoku puzzles. For instance, exact methods such as constraint programming [2–4] and boolean satisfiability [5] are well-known candidates for the efficient handling of such kind of puzzles. In the context of approximate methods, genetic programming [1] and metaheuristics in general [6–10] have illustrated promising results. Additional, but less traditional, Sudoku solving techniques have been proposed as well, such as Sinkhorn balancing [11], rewriting rules [12], and entropy minimization [13].

In this paper, we focus on approximate methods. We propose a new algorithm for the efficient solving of Sudoku instances based on cuckoo search, geometric operators, and prefiltering phases. The cuckoo search is a relatively modern nature-inspired metaheuristic [14–18] to which we introduce geometric operators in order to correctly move to promising regions of a discrete space of solutions. This combination is additionally enhanced with a domain reducer component based on local consistencies. The idea is to previously delete from the search space the values that do not conduct to any feasible solution. This integration straightforwardly alleviates the work of the metaheuristic leading to a faster solving process. We illustrate encouraging experimental results where our approach noticeably competes with the best approximate methods reported in the literature.

This paper is organized as follows. In Section 2, we describe the previous work.  Section 3 presents the classic cuckoo search algorithm. The geometric operators and the prefiltering phase employed are illustrated and exemplified in Sections 4 and 5, respectively. The resulting new cuckoo search algorithm is presented in Section 6, followed by the corresponding experimental results. Finally, in Section 8, we conclude and give some directions for future work.

## 2. Related Work

Sudoku puzzles have been solved with various techniques during the last decades. For instance, complete methods such as Boolean satisfiability and constraint satisfaction can clearly be used to solve Sudokus [3–5, 19]. In this paper, we focus on incomplete search methods, specially on solving hard instances of such a puzzle. Within this scenario, different solutions have been suggested, mainly based on metaheuristics. For instance, Lewis [7] models the puzzle as an optimization problem where the number of incorrectly placed digits on the board must be minimized. The model is solved by using simulated annealing, but the approach is mostly focused on producing valid Sudokus than on the performance of the resolution. In [10], an ant colony algorithm is proposed, where the problem is modeled in an opposite form: maximizing the number of correctly filled cells. The best result completes only 76 out of 81 cells of the puzzle. In [8], a particle swarm optimizer (PSO) for solving Sudokus is presented, but the goal of authors was rather to validate the use of geometric operators in PSO for complex combinatorial spaces.

In [6], a hill-climbing algorithm for Sudokus is reported. The approach succeeds in solving easy Sudoku instances, failing for medium and hard ones. In the same work, a genetic algorithm (GA) outperforms the hill-climber previously presented. Such a GA is tuned with geometric operators, in particular Hamming space crossovers and swap space crossovers, reporting solutions for a hard Sudoku. In Mantere and Koljonen [1], another GA is proposed that succeeds for easy and medium instances, but it only reaches the optimum in 2 out of 30 tries for a hard Sudoku. A cultural algorithm is proposed by the same authors [9], but it is generally outperformed by the GA previously reported. In Soto et al. [20], a tabu search is tuned with a prefiltered phase, being capable of solving 30 out of 30 tries for a hard Sudoku.

## 3. Cuckoo Search

Cuckoo search is a nature-inspired metaheuristic, based on the principle of the brood parasitism of some cuckoo species. This kind of bird has an aggressive reproduction strategy, which is based on the use of foreign nests for incubation. Cuckoos proceed by laying their eggs in nests from other bird species, removing the other bird eggs to increase incubation probability. Eventually, cuckoo eggs may be discovered by the host bird, which might act in two ways: taking off the alien eggs or simply abandoning its nest and building a new one elsewhere. In practice, an egg represents a solution and cuckoo eggs represent potentially better solutions than the current ones in nests. In the simplest form each nest has only one egg.

The cuckoo search procedure for minimization is described in Algorithm 1. The process begins by randomly generating an initial population of host nests. Next, the algorithm iterates until a given stop criterion is reached, which is commonly a maximum number of iterations. At line 3, a new solution is created, normally by employing a random walk via Lévy flights. Equation (1) describes such a random walk, where  *x*_*i*_^*t*+1^ is the new solution,  *t*  corresponds to the iteration number, and the product ⊕ means entrywise multiplications. The  *α*  parameter is the step size, where  *α* > 0, and determines how far the process can go for a fixed number of iterations. Then, at line 4, an  *Egg*_*j*_  is randomly chosen to be then compared with the previous one in order to keep the egg exhibiting the best cost. Finally, the worse nests are abandoned depending on the probability  *p*_*a*_  and new solutions are built:

(1) $$\begin{array}{c} x_{i}^{t+1}=x_{i}^{t}+\alpha ⊕\text{L}\overset{´}{\text{e}}\text{vy}\left(\lambda \right),\\ \text{L}\overset{´}{\text{e}}\text{vy}\sim u=t^{-\lambda },\;\left(1<\lambda \leq 3\right). \end{array}$$

## 4. Geometric Operators

The cuckoo search has been originally designed for continuous domains, while Sudokus own discrete values. Then, a discretization phase for the CS algorithm is mandatory to correctly explore the potential solutions. The discretization phase applied here has been inspired from the work reported in [8], where a particle swarm optimization algorithm is adapted to solve discrete domains. The idea relies on the use of geometric-based operators able to correctly move to promising regions of a discrete search space. In particular, for this work, we employ the partially matched crossover, the geometric crossover, and the feasible geometric mutation, which are described in the following.

### 4.1. Partially Matched Crossover

The partially matched crossover (PMX) basically works with two parents, creating two crossover points that are selected at random and then PMX proceeds by position swap. The PMX process is described in Algorithm 2.

At the beginning, a segment from *Parent*_1_ is randomly selected and copied to the child. Then, looking in the same segment positions in *Parent*_2_, each value not copied to the child is stored in a list. Then, for each value in this list, the **V** value is located in *Parent*_1_ in the position given by the index of  *val*  in *Parent*_2_. Then, an if-else conditional operates as follows: if the index of **V** is present in the original segment, **V** becomes the new  *val*  and the process goes to line 4; otherwise,  *l* *val*  is inserted into the child in the position given by the index of **V** in *Parent*_2_. Finally, the remaining positions from *Parent*_2_ are copied to the child. The PMX operator applied to the Sudoku can be seen in Example 1.

**Example 1 (PMX crossover)**

Let two rows located in the same position from different solutions of a Sudoku instance be the parents. The corresponding child produced by the PMX crossover is constructed as follows.

(1) A random segment of consecutive digits from *Parent*_1_ is copied to the child. Assuming that 1 corresponds to the index of the first position, the segment has size 5, from index 4 to 8:
  (2) Parent1:Parent2:Child:847912---36251345673625198-
(2) “4” is the first value in the observed segment of  *Parent*_2_  that is not present in the child. Then,  *val* = 4, and the index of  *val*  in *Parent*_2_ is “5.” Hence, the **V** value corresponds to “6.” Next, the index of **V** in *Parent*_2_ is “7.” This index exists in the observed segment, so the process comes back to line 4 using “6” as  *val*:
  (3) Parent1:Parent2:Child:847912---36251345673625198-
(3) Now, using “6” as  *val*, the new **V** is “5.” Then, the index of “5” in *Parent*_2_ also appears within the segment. So, the process comes back again to line 4 using “5” as  *val*:
  (4) Parent1:Parent2:Child:847912---36251345673625198-
(4) Then, the **V** value is “2,” and its index in *Parent*_2_ does not appear within the segment. Hence, we obtain a position in the child for the value “4” from step 2:
  (5) Parent1:Parent2:Child:847912--436251345673625198-
(5) “7” is the next value from *Parent*_2_ in the segment that is not already included in the child. Then, “1” is the **V** value, whose index does not appear within the segment as well. Hence, a position for the value “7” is obtained in the child:
  (6) Parent1:Parent2:Child:847912-7436251345673625198-
(6) Now, everything else from *Parent*_2_ is copied down to the child:
  (7) Parent1:Parent2:Child:847912974362513456736261988

### 4.2. Multiparental Sorting Crossover

This operator may employ multiple parents; our approach based on [8] uses three, where each one represents a row of a potential solution: one from the best solution of all generations, one from the best solution of the current generation, and one from the current solution. Each parent is associated with a weight (*w*) according to (8) in order to control the relevance of each solution in the generation of the new one. The influence of parents given by the weights is reflected in a mask used in the process.

The multiparental crossover is described in Algorithm 3. The three parents and the mask are the input of the procedure, and the child resulting from the crossover is the output. A for each loop is used to scan the mask, where every entry indicates which parent the other two parents need to be equal to for that specific position. The replacement depends on the value of the mask and it is performed by swapping the corresponding values as indicated in the conditionals stated at lines 2, 6, and 10. The swapping process is described in Algorithm 4:

(8) $$\begin{array}{c} w_{1}+w_{2}+w_{3}=1,\;\;\text{such}\;\text{that}\;\;w_{i}>0\;\forall i\in \left\{1,2,3\right\}. \end{array}$$

**Example (multiparental sorting crossover)**

Let *Parent*_1_ be a row from the best solution of all generations, *Parent*_2_ a row from the best solution of the current generation, and *Parent*_3_ a row from the current solution. We employ 0.55 as the weight for *Parent*_1_, 0.33 for *Parent*_2_, and 0.12 for *Parent*_3_. Those weights represent the percentage of appearances of the given parent within the mask. For instance, *Parent*_1_  appears five times in the mask, *Parent*_2_ three times, and *Parent*_3_  once. The corresponding child produced by the multiparental sorting crossover is constructed as follows.

(1) A mask of parent length is randomly generated according to the parent weights:
  (9) Mask:Parent1:Parent2:Parent3:312111122847362519912345678479362518
(2) The first value of the mask corresponds to parent “3”, and then the first value of  *Parent*_1_  and  *Parent*_2_  needs to be equal to the first value of  *Parent*_3_, which is “4.” To this end, in  *Parent*_1_  and  *Parent*_2_, the first cell is swapped with the cell that holds the value “4”:
  (10) Mask:312111223Parent1:4_8_7362519Parent2:4_1239_5678Parent3:479362518Child4
(3) Next, the second value from the mask is “1.” The swapping process is analogous:
  (11) Mask:312111223Parent1:487362519Parent2:48_2395671_Parent3:48_9362517_Child48
(4) Following the same procedure, the last step is shown below obtaining 4 8 2 3 6 7 9 5 1 as the new child:
  (12) Mask:312111223Parent1:482367951Parent2:482367951Parent3:482367951Child482367951

### 4.3. Feasible Geometric Mutation

This is a simple operator used to maintain diversity in the solutions. It swaps two nonfixed elements in a row guaranteeing that mutation is applied only over the cells with no given value. The procedure is described in Algorithm 5.

**Example 3 (feasible geometric mutation)**

Let us consider a given Sudoku row and a solution candidate row, as shown below:

(13) Sudoku  problem  row:---3-----Solution  candidate  row:974362518

The mutation is allowed in any cell except for cell 4, which owns the value 3 as given for the Sudoku instance. Examples of allowed and forbidden mutations are depicted below:

(14) allowed  mutation:964372518forbidden  mutation:974462518

## 5. Prefiltering Phase

In the presence of unfeasible solutions, the cuckoo procedure is responsible for detecting and discarding them in order to conduct the search to feasible regions of the space of solutions. The goal of the prefiltering phase is to alleviate the work of the cuckoo algorithm by previously eliminating those unfeasible values. This is possible by representing the Sudoku as a constraint network [4] and then applying efficient filtering techniques from the constraint satisfaction domain. In this context, arc-consistency [2] is a widely employed local consistency for filtering algorithms. Arc-consistency was initially defined for binary constraint [21, 22]. We employ here the more general filtering for nonarbitrary constraints named generalized arc-consistency (GAC). The idea is to enforce a local consistency to the problem in a process called constraint propagation. Before detailing this process, let us introduce some necessary definitions [23].

**Definition 4 (constraint)**

A constraint  *c*  is a relation defined on a sequence of variables  *X*(*c*) = (*x*_*i*_1__,…, *x*_*i*_|*X*(*c*)|__), called the scheme of  *c*; *c*  is the subset of  *ℤ* ^|*X*(*c*)|^  that contains the combinations of tuples  *τ* ∈ *ℤ* ^|*X*(*c*)|^  that satisfy  *c*.  |*X*(*c*)|  is called the arity of  *c*. A constraint  *c*  with scheme  *X*(*c*) = (*x*_1_,…, *x*_*k*_)  is also noted as  *c*(*x*_1_,…, *x*_*k*_).

**Definition 5 (constraint network)**

A constraint network also known as constraint satisfaction problem (CSP) is defined by a triple  *N* = 〈*X*, *D*, *C*〉, where

- *X*  is a finite sequence of integer variables  *X* = (*x*_1_,…, *x*_*n*_);
- *D*  is the corresponding set of domains for  *X*; that is,  *D* = *D*(*x*_1_) × ⋯×*D*(*x*_*n*_), where  *D*(*x*_*i*_) ⊂ *ℤ*is the finite set of values that variable  *x*_*i*_  can take;
- *C*  is a set of constraints  *C* = {*c*_1_,…, *c*_*e*_}, where variables in  *X*(*c*_*j*_)  are in  *X*.

**Example (the Sudoku as a constraint network)**

Let  〈*X*, *D*, *C*〉  be the constraint network, which is composed of the following.

- *X* = (*x*_1,1_,…, *x*_*n*,*m*_)  is the sequence of variables, and  *x*_*i*,*j*_ ∈ *X*  identifies the cell placed in the *i*th row and *j*th column of the Sudoku matrix, for  *i* = 1,…, *n*  and  *i* = 1,…, *m*.
- *D*  is the corresponding set of domains, where  *D*(*x*_*i*__*j*_) ∈ *D*  is the domain of the variable  *x*_*ij*_.
- *C*  is the set of constraints defined as follows.
  - To ensure that values are different in rows and columns, *x*_*k*,*i*_ ≠ *x*_*k*,*j*_∧*x*_*i*,*k*_ ≠ *x*_*j*,*k*_, for all (*k* ∈ [1,9], *i* ∈ [1,9], *j* ∈ [*i* + 1,9]).
  - To ensure that values are different in subgrid: *x*_(*k*1−1)∗3+*k*2,(*j*1−1)∗3+*j*2_ ≠ *x*_(*k*1−1)∗3+*k*3,(*j*1−1)∗3+*j*3_, for all (*k*1, *j*1, *k*2, *j*2, *k*3, *j*3 ∈ [1,3] | *k*2 ≠ *k*3∧*j*2 ≠ *j*3).

**Definition 7 (projection)**

A projection of  *c*  on  *Y*  is denoted as  *π*_*Y*(*c*)_, which defines the relation with scheme  *Y*  that contains the tuples that can be extended to a tuple on  *X*(*c*)  satisfying  *c*.

**Definition 8 ((generalized) arc-consistency)**

Given a network  *N* = 〈*X*, *D*, *C*〉, a constraint  *c* ∈ *C*, and a variable  *x*_*i*_ ∈ *X*(*c*),

- a value  *v*_*i*_ ∈ *D*(*x*_*i*_)  is consistent with  *c* ∈ *D*  if and only if there exists a valid tuple  tau  satisfying  *c*  such that  *v*_*i*_ = *τ*[{*x*_*i*_}]; such a tuple is called a support for  (*x*_*i*_, *v*_*i*_)  on  *c*;
- the domain  *D*  is (generalized) arc-consistent on  *c*  for  *x*_*i*_  if and only if all the values in  *D*(*x*_*i*_)  are consistent with  *c*  in *D*; that is, *D*(*x*_*i*_)⊆*π*_*x*_*i*__(*c*∩*π*_*X*(*c*)_(*D*));
- the network  *N*  is (generalized) arc-consistent if and only if  *D*  is (generalized) arc-consistent for all variables in  *X*  on all constraints in  *C*.

The filtering process is achieved by enforcing the arc-consistency on the problem. This can be carried out by using Algorithms 6 and 7. The idea is to revise the arcs (the constraint relation between variables) by removing the values from  *D*(*X*_*i*_)  that lead to inconsistencies with respect to a given constraint. Such a revision process is done by Algorithm 6, which takes each value  *v*_*i*_ ∈ *D*(*x*_*i*_)  (line 2) and analyses the space  *τ* ∈ *c*∩*π*_*X*(*c*)_(*D*), searching for a support on constraint  *c*  (line 3). If support does not exist, the value  *v*_*i*_  is eliminated from  *D*(*x*_*i*_). Finally, the procedure informs if the domain has been modified by returning the corresponding Boolean value (line 8).

The role of Algorithm 7 is to guarantee that every domain is arc-consistent. This is done by iteratively revising the arcs by performing calls to Algorithm 6. At the beginning, a list called  *Q*  is filled with pairs (*x*_*i*_, *c*) such that  *x*_*i*_ ∈ *X*(*c*). Pairs for which  *D*(*x*_*i*_)  is not ensured to be arc-consistent are kept in order to avoid useless calls to Algorithm 7. This is a main advantage of AC3 with respect to its predecessor Algorithms AC1 and AC2. Then, at line 2, a while statement controls the calls to Revise3. If a true value is received from Revise3,  *D*(*X*_*i*_)  is verified and if no value remains within the domain, the algorithm returns false. If there still exist values in  *D*(*X*_*i*_), normally, a value for another variable  *x*_*j*_  has lost its support on  *c*. Hence, the list  *Q*  must be refilled with all pairs (*x*_*i*_; *c*). The process finishes and returns true when all remaining values within domains are arc-consistent with respect to all constraints.

**Example (enforcing AC3 on a Sudoku puzzle)**

**Figure 2:**
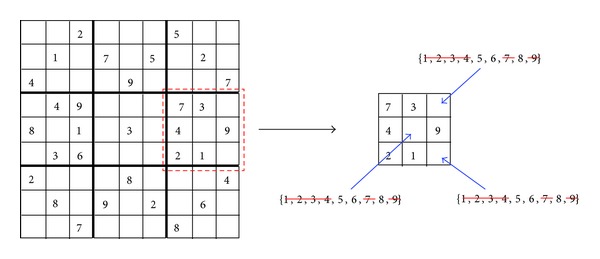
Enforcing AC3 to a subgrid constraint.

Let us exemplify the work of the prefiltering phase by enforcing the AC3 on three constraints of a Sudoku instance. We begin by enforcing the AC3 on a given subgrid (enclosed with dashed lines in Figure 2) of the Sudoku puzzle. The subgrid has three variables with no value assigned (*x*_4,9_, *x*_5,8_, and  *x*_6,9_). Then, enforcing AC3 via the GAC3 algorithm with respect to the subgrid constraint (second constraint from Example 6) leads to the elimination of six values from  *D*(*x*_4,9_), *D*(*x*_5,8_), and  *D*(*x*_6,9_). Those values have no support on the verified constraint; that is, they have been already taken for another cell on the same subgrid. Thus, the original domains for the three variables are reduced to  {5,6, 8}.

**Figure 3:**
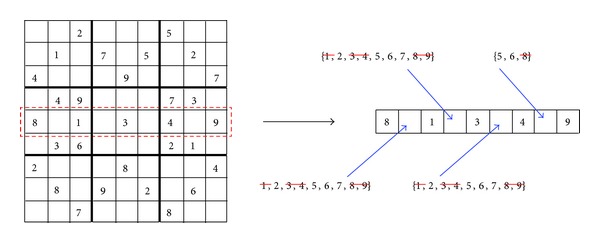
Enforcing AC3 to a row constraint.

**Figure 4:**
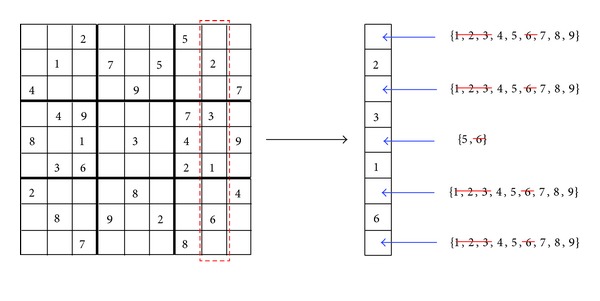
Enforcing AC3 to a column constraint.

In Figure 3, the AC3 is enforced to a row of the puzzle. This row has four variables (*x*_5,2_, *x*_5,4_, *x*_5,6_, and  *x*_5,8_) with no assigned value. The GAC3 algorithm filters from domains five values with no support from  *D*(*x*_5,2_), *D*(*x*_5,4_), and  *D*(*x*_5,6_). The variable  *x*_5,8_  has been refiltered, remaining only two possible values. Finally, in Figure 4, four values are filtered from four variables, remaining only one possible value for variable  *x*_5,8_.

## 6. The Prefiltered Cuckoo Search via Geometric Operators

**Figure 5:**
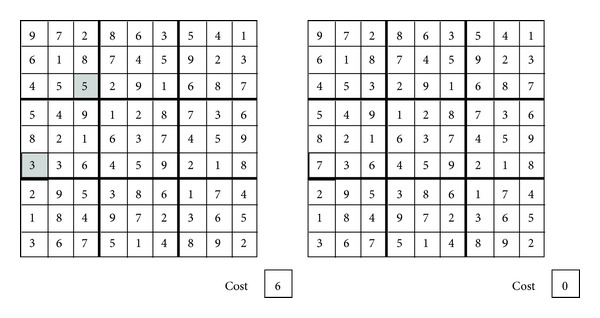
Solution cost of a Sudoku puzzle.

The cuckoo search proposed here combines geometric operators with prefiltering phases. The goal is to enhance the performance of the cuckoo search as well as to allow it to correctly explore a discrete search space. Algorithm 5 illustrates the new hybrid algorithm. Now the input set is quite larger. It considers the size of the nest, the constraint network  〈*X*, *D*, *C*〉  representing the Sudoku problem, and two parameters that define probabilities for regulating the usage of geometric operators. As output, the procedure returns the best egg reached by the algorithm. The prefiltering phase via the AC3 algorithm is triggered at the beginning. The AC3 algorithm receives as input the constraint network  〈*X*, *D*, *C*〉  of the Sudoku and reduces if possible the set of domains  *D*  by deleting the unfeasible values. Then, an initial population of nests is generated but, unlike the classic cuckoo, the generation is bounded to the reduced set of domains  *D*. Between lines 3 and 13, a while loop manages the iteration process until the stop condition is reached, which for the current implementation corresponds to a maximum number of iterations. At line 4, an *Egg*_*i*_ is randomly chosen from nests to which the geometric operators are then applied. The usage of geometric operators is illustrated in Algorithm 9, where they apply only whether the evaluated egg is not the best one. The PMX and multiparent sorting crossovers act depending on a random value and on the  *P*_*pmx*-*multi*_  probability. Analogously, the mutation operates using the  *P*_*mutate*_  probability, and  *D*  is used as input of the mutation to validate that only feasible mutations are carried out. At line 6, an  *Egg*_*j*_  is randomly chosen to be then compared with the previous one in order to keep the one exhibiting the best cost. The cost of a solution corresponds to the sum of wrong values in subgrids, columns, and rows (see Figure 5). At line 11, the worse nests are abandoned depending on probability  *p*_*a*_. Finally, new solutions are built, but again, the set of filtered domains  *D*  is considered in order to avoid unfeasible solutions.  Algorithm 8 illustrates the prefiltered discrete cuckoo search.

## 7. Experimental Results

Different experiments have been performed in order to validate our approach. The Sudoku benchmarks used have been taken from [24], which are organized in three difficulty levels: easy, medium, and hard. All tested Sudokus have a unique solution. The algorithms have been implemented in Octave 3.6.3, and the experiments have been performed on a 2.0 GHz Intel Core2 Duo T5870 with 1 Gb RAM running Fedora 17. The configuration of the proposed cuckoo search is the following, which corresponds to the best one achieved after a tuning phase:  *P*_*a*_ = 0.25, *P*_*pmx*-*multi*_  = 0.7, *P*_*mutate*_ = 0.9, and  *Nest*_*size*_ = 10.

**Figure 6:**
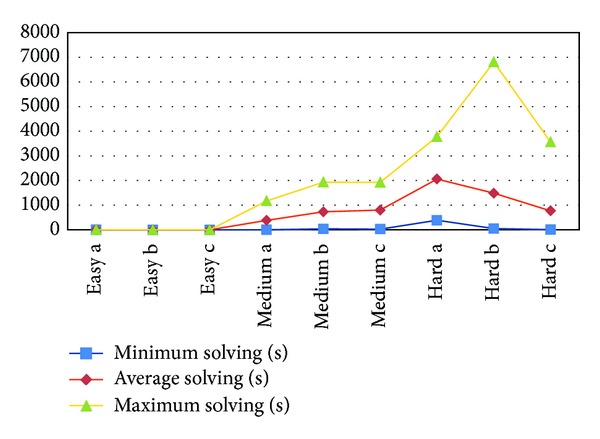
Comparing solving times for Sudoku.

Table 1 illustrates the results of solving 9 problems, 3 from each difficulty level, by using the proposed cuckoo search algorithm considering 10000 iterations. From left to right, the table states the number of tries performed, the number of tries solved, the minimum solving time reached, the average solving time, the maximum solving time, and the standard deviation (SD). Regarding the easy Sudokus, the proposed cuckoo search is able to rapidly solve them reaching a 100% of success (50 out of 50 tries solved). Then, increasing one difficulty level, the percentage of success shortly diminishes, keeping the 100% of success for the “medium a” benchmark. Finally, for hard Sudokus, the runtime naturally gets bigger (see Figure 6); however, the performance is reasonable, reaching a 51% (50 out of 97 tries solved) of success for “hard a,” 80% (50 out of 62 tries solved) of success for “hard b,” and a 70% (50 out of 71 tries solved) of success for “hard c.”

In Table 2, the performance of the proposed cuckoo search is compared with the best-performing incomplete methods reported in the literature: a genetic algorithm (GA) [1] and a hybrid tabu search (hybrid TS) [20]. We contrast the number of problems solved from a total of 30 tries taking into account both unlimited iterations and 100000 iterations. The results depict that the three techniques are able to easily succeed for the first Sudoku level. In the presence of medium Sudokus, the GA begins to decrease its performance, being able to solve a medium Sudoku with a 33% of success (10 out of 30 tries solved). The cuckoo search and the hybrid TS keep their 100% of success. Finally, observing hard Sudokus, the performance of the cuckoo search and the hybrid TS is considerably better than GA, which solves only 2 out of 30 tries, while our proposal as well as the hybrid TS is able to reach a 100% of success.

## 8. Conclusions and Future Work

In this paper, we have presented a prefiltered cuckoo search algorithm tuned with geometric operators. The geometric operators allow one to drive the search to promising regions of a discrete space of solutions, while the prefiltering phase attempts to previously delete from domains the values that do not conduct to any feasible solution. In this way, the work of the metaheuristic is alleviated leading to a faster solving process. We have performed a set of experiments in order to compare our approach with the best-performing approximate methods reported in the literature. We have considered Sudokus from different difficulty levels: easy, medium, and hard. In the presence of easy Sudokus, the proposed cuckoo search is able to reach a 100% of success. When solving medium difficulty Sudokus, the cuckoo search keeps its 100% of success, while the best GA reported is able only to solve 10 out of 30 tries. Finally, regarding hard Sudokus, the cuckoo search noticeably competes against the best incomplete method reported for Sudokus, both reaching a 100% of success considering 100000 iterations.

We visualize different directions for future work; perhaps the clearest one is the introduction of prefiltering phases to additional metaheuristics such as particle swarm optimization, ant, or bee colony algorithms to solve Sudokus or any combinatorial problem. Evaluating the behaviour of geometric operators in other swarm-based metaheuristics is another interesting work to develop. Finally, the use of autonomous search [25–28] for the self-tuning of a metaheuristic interacting with prefiltering phases will be also an appealing research direction to pursue.

## Figures and Tables

**Algorithm 1:**
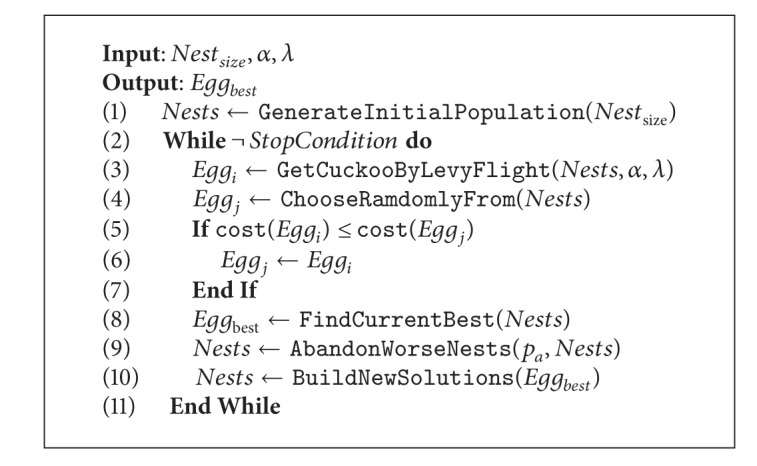
Cuckoo search.

**Algorithm 2:**
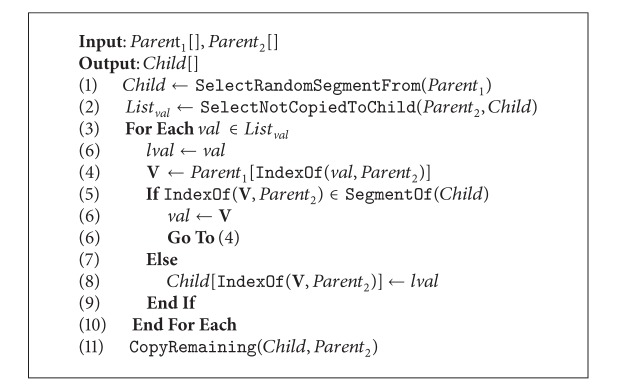
PMX crossover.

**Algorithm 3:**
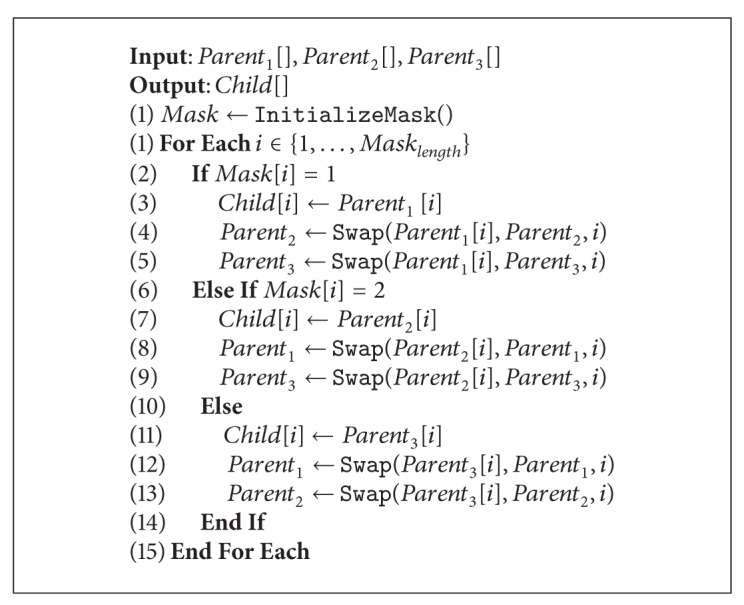
Multiparental sorting crossover.

**Algorithm 4:**
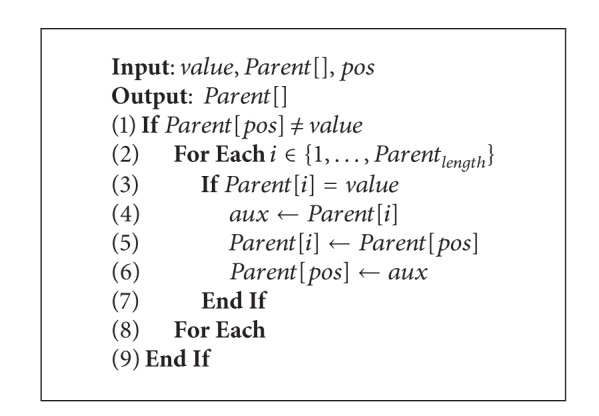
Swap.

**Algorithm 5:**
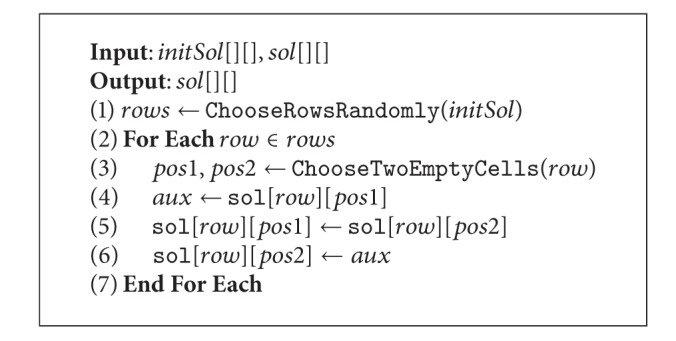
Feasible geometric mutation.

**Algorithm 6:**
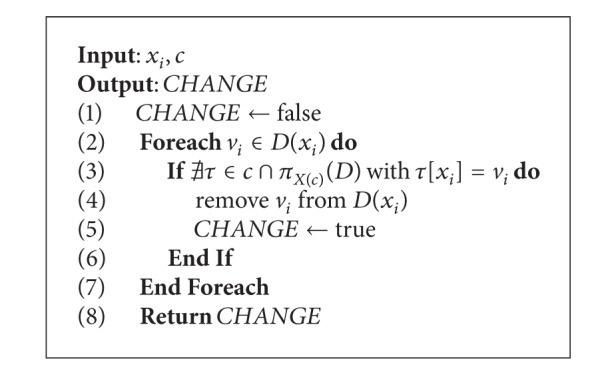
Revise3.

**Algorithm 7:**
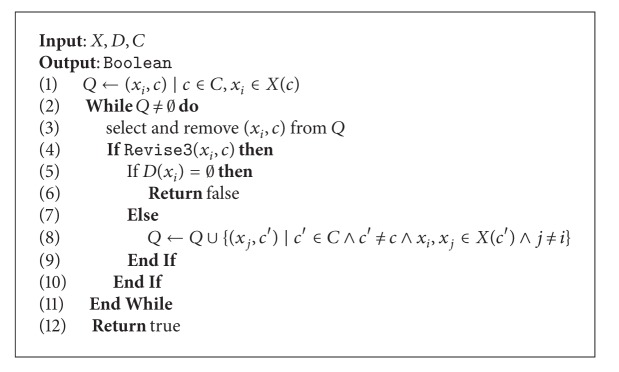
AC3/GAC3.

**Algorithm 8:**
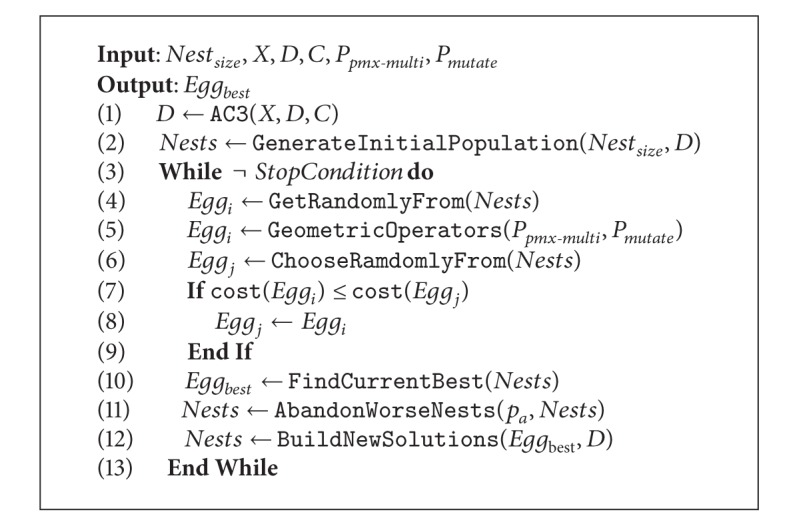
Prefiltered discrete cuckoo search.

**Algorithm 9:**
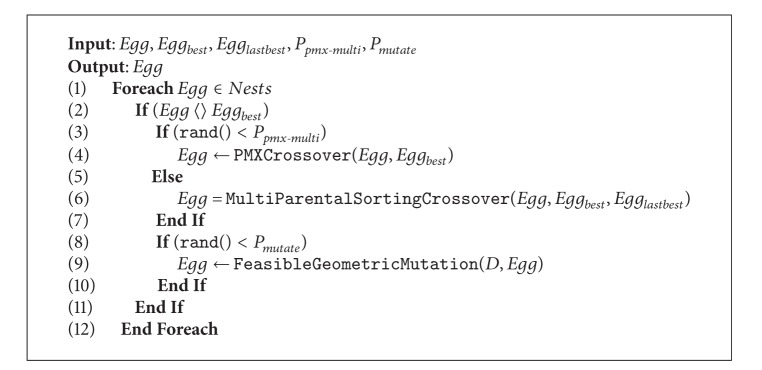
Geometric operators.

**Table 1:** Solving Sudokus with the prefiltered discrete cuckoo search considering 10000 iterations.

	Tries	Solved	Min. solving time (sec)	$\bar{x}$ solving time (sec)	Max. solving time (sec)	SD time (sec)
Easy a	50	50	1.302	1.310	1.329	0.004
Easy b	50	50	1.005	1.007	1.029	0.003
Easy c	50	50	1.019	1.021	1.036	0.003
Medium a	50	50	2.501	384.341	1171.312	291.336
Medium b	84	50	38.32	729.673	1932.691	516.867
Medium c	67	50	26.807	800.471	1923.442	561.877
Hard a	97	50	385.652	2059.690	3777.340	984.921
Hard b	62	50	49.608	1484.692	6810.882	1473.731
Hard c	71	50	9.497	771.211	3561.042	825.516

**Table 2:** Comparing the prefiltered discrete cuckoo search with the best-performing incomplete methods for Sudokus considering 100000 and unlimited iterations.

Problem	Prefiltered discrete CS		Hybrid TS		GA	
	Unlimited iterations	100000 iterations	Unlimited iterations	100000 iterations	Unlimited iterations	100000 iterations
Easy a	30	30	30	30	30	29
Easy b	30	30	30	30	30	30
Easy c	30	30	30	30	30	30
Medium a	30	30	30	30	30	10
Medium b	30	30	30	30	—	—
Medium c	30	30	30	30	—	—
Hard a	30	30	30	30	30	2
Hard b	30	30	30	30	—	—
Hard c	30	30	30	30	—	—
